# The Transcriptome of *Exophiala dermatitidis* during *Ex-vivo* Skin Model Infection

**DOI:** 10.3389/fcimb.2016.00136

**Published:** 2016-10-24

**Authors:** Caroline Poyntner, Barbara Blasi, Elsa Arcalis, Ursula Mirastschijski, Katja Sterflinger, Hakim Tafer

**Affiliations:** ^1^Department of Biotechnology, VIBT EQ Extremophile Center, University of Natural Resources and Life SciencesVienna, Austria; ^2^Department for Applied Genetics and Cell Biology, Molecular Plant Physiology and Crop Biotechnology, University of Natural Resources and Life SciencesVienna, Austria; ^3^Klinikum Bremen-Mitte, Department of Plastic, Reconstructive and Aesthetic Surgery, Faculty of Biology and Chemistry, Center for Biomolecular Interactions Bremen, University BremenBremen, Germany

**Keywords:** RNA sequencing, black yeast, skin infection, virulence, skin model, fungal pathogens

## Abstract

The black yeast *Exophiala dermatitidis* is a widespread polyextremophile and human pathogen, that is found in extreme natural habitats and man-made environments such as dishwashers. It can cause various diseases ranging from phaeohyphomycosis and systemic infections, with fatality rates reaching 40%. While the number of cases in immunocompromised patients are increasing, knowledge of the infections, virulence factors and host response is still scarce. In this study, for the first time, an artificial infection of an *ex-vivo* skin model with *Exophiala dermatitidis* was monitored microscopically and transcriptomically. Results show that *Exophiala dermatitidis* is able to actively grow and penetrate the skin. The analysis of the genomic and RNA-sequencing data delivers a rich and complex transcriptome where circular RNAs, fusion transcripts, long non-coding RNAs and antisense transcripts are found. Changes in transcription strongly affect pathways related to nutrients acquisition, energy metabolism, cell wall, morphological switch, and known virulence factors. The L-Tyrosine melanin pathway is specifically upregulated during infection. Moreover the production of secondary metabolites, especially alkaloids, is increased. Our study is the first that gives an insight into the complexity of the transcriptome of *Exophiala dermatitidis* during artificial skin infections and reveals new virulence factors.

## 1. Introduction

Black yeasts are a special group of Ascomycetes characterized by melanized cells and the ability to form hyphal and yeast-like budding states. They are known for their capacity to survive in extreme habitats, which range from hot and cold deserts, bare rock surfaces, glaciers (Sterflinger et al., 2012; Selbmann et al., 2015), contaminated soils and rivers. They also thrive in man-made extreme environments like dishwashers or sauna facilities (Matos et al., 2002; Gümral et al., 2016). In both immunocompetent and immunocompromised humans, black yeasts can colonize the skin and bones as well as the lymphatic and nervous systems (Seyedmousavi et al., 2014). A serious and life-threatening problem can be fungal wound infections in burn patients with large total charred body surface areas or compromised immune response (Schaal et al., 2015). The clinical picture caused by black yeasts includes mere superficial blackening of the skin, for example Tinea nigra caused by *Hortea werneckii*, necrotic lesions of the skin and formation of cysts, granuloma and tumors, eumycetoma and chromoblastomycosis triggered by species of *Cladophialophora, Fonsecea* and *Exophiala*, to deep systemic infection of the lymphoid system and passage of the blood brain barrier with fatal lesions in the human brain, e.g., provoked by species of *Cladophialophora* and *Exophiala*. Black yeasts are involved in a broad range of diseases and detailed knowledge of the ways of infection, the factors leading to virulence and outbreak of the diverse clinical pictures are still elusive.

Since 1990, an increasing number of phaeohyphomycosis, i.e., infections caused by dematiaceous or pigmented filamentous fungi that contain melanin in their cell wall, has been reported in immunocompromised patients (Revankar et al., 2002) and the list is expected to grow. This is not only explained by the availability of better diagnostic tools (Seyedmousavi et al., 2014) but particularly by the outstanding evolutionary diversification and adaptation of black yeast to their mammalian hosts. Especially the genus *Exophiala* can be regarded as an evolutionary hotspot with a high diversification and emerging adaptation toward many environments (de Hoog et al., 2003). *Exophiala dermatitidis* in particular is a polyextremophilic, poikilohydric and poikilotrophic fungus tolerating low and high temperatures, wateravailability and pH values. As a consequence the fungus is commonly found in a wide range of natural and anthropogenic environments. In fact steam bath facilities, saunas and dishwashers are man-made extreme environments where *Exophiala dermatitidis* is thriving (Matos et al., 2002; Gostinčar et al., 2011; Zalar et al., 2011).

Infection with *Exophiala dermatitidis* may affect cutaneous and subcutaneous regions, leading among other to otitis externa, keratitis and onychomycosis (Matsumoto et al., 1993; de Hoog, 2000). Colonization might also happen in the lung of cystic fibrosis patients, which represent 2–8% of the susceptible patient population (Horré et al., 2004; Chotirmall and McElvaney, 2014), or the intestinal tract (de Hoog et al., 2005). *Exophiala dermatitidis* is also the etiologic agent of life threatening systemic infections that are predominantly found in patients with diabetes mellitus, rheumatic arthritis, lymphocytic leukemia or of Asian descent (Sudhadham et al., 2008). While the incidence of infection with *Exophiala dermatitidis* is low, its mortality rate for systemic infections of 40% is high (Chen et al., 2016). In case of neurotrophic infection the fatality rate has been reported to be over 80% (Patel et al., 2013).

The principles of pathogenesis, the host response as well as the difference in incidence in humans of different etiology and predisposition are poorly understood. The mechanisms sustaining the polyextremophily of *Exophiala dermatitidis* are both involved in the pathogenicity and the antifungal resistance of this black yeast (Seyedmousavi et al., 2014). Melanin, which is not essential for growth and development, plays a crucial role in virulence and pathogenicity, allows the fungus to escape phagocytosis and protects him against free radicals (Paolo et al., 2006; Revankar and Sutton, 2010). Thermotolerance, cellular plasticity and the ability to assimilate aromatic hydrocarbons are features that emerged in order to adapt to harsh environments and that are used by *Exophiala dermatitidis* to successfully infect and invade its host (Ye and Szaniszlo, 2000; Abramczyk et al., 2009; Seyedmousavi et al., 2014).

*Exophiala dermatitidis* has become the most studied species within the group of black yeasts due to its human pathogenicity, its polyextremophily, its association to the human environment as well as its close relation to many other black fungi. While studies looking at the transcriptome or the proteome have been released recently, their focus was limited to fungal response under pH and temperature stress (Chen et al., 2014; Blasi et al., 2015; Tesei et al., 2015). This is in contrast to other pathogenic fungi where transcriptomes studies of the infection are available both for the host and the pathogen (Enguita et al., 2016).

In this work we present for the first time results of an artificial traumatic infection of human skin models by *Exophiala dermatitidis*. *Ex-vivo* human skin explants were wounded and inoculated with *Exophiala dermatitidis* and incubated at 37°C to simulate human body temperature for 1 week. At the end of the experiment, attachment and fungal growth on the skin grafts were confirmed microscopically and biomass was harvested for RNA sequencing. These data, together with multiple genomes alignments, were used to improve the current *Exophiala dermatitidis* annotation (Chen et al., 2014) by identifying new UTRs, adding splicing variants and reporting new coding and (long) non-coding elements. Transcriptome data from *Exophiala dermatitidis* grown on a nylon membrane in cell culture medium were used to find new coding and non-coding transcript, improve the *Exophiala dermatitidis* genome annotation and identify differentially expressed coding and non-coding genes. We observe differential regulation of known virulence factors as well as newly annotated coding and non-coding genes. These findings will help to better understand which mechanisms are employed by *Exophiala dermatitidis* to infect skin.

## 2. Materials and methods

### 2.1. Fungal strain, skin grafts, and culture conditions

Skin for the *ex-vivo* skin wound model was obtained from two healthy patients (60 and 38 years) undergoing breast reduction from the Department of Plastic, Reconstructive and Aesthetic Surgery, Klinikum Bremen-Mitte, Germany. Skin was either a full thickness graft or a partial thickness graft. Skin stripes were cut into 5 × 5 cm pieces and on the surface cuts were done with a scalpel to simulate wounds. On top of each wound *Exophiala dermatitidis* (CBS 525.76) was inoculated with a sterile inoculation loop taken from a 7 days old culture grown on malt extract agar (2% malt extract, 2% D-glucose, 0.1% bacto-peptone and 2% agar). The skin was cultured as described by Mirastschijski et al. (2002): Culture media composed of DMEM (4500 mg/L Glucose, D5671, Sigma Aldrich, St. Louis, MO), Penicillin—Streptomycin (100 U Penicillin per mL, 100 μg Streptomycin, P4333 Sigma Aldrich, St. Louis, MO) and 10% v/v heat-inactivated bovine serum (Gibco, Life Technologies Carlsbad, CA) was carefully added to the skin grafts. A surface-liquid interface was built, allowing the skin to float in media but keeping the surface unsubmerged to prevent the inoculum from being washed away. The cultures were kept at 37°C for 7 days and medium was exchanged every second day. For every part and patient a control without fungal inoculum was kept. As negative control, *Exophiala dermatitidis* was inoculated on a prewetted Nylon membrane (Whatman 0.45 μm) for 2 days at 37°C. Culture medium, as described, was added and kept for 1 week at 37°C. *Exophiala dermatitidis* cells growing on the skin models were collected by scrapping them from the skin surface.

### 2.2. Ethics statement

The use of human skin for this study was approved by the ethics committee of the Medical Council of Bremen (No 336-2012). Written consent was obtained from all donors prior to the operation.

### 2.3. Microscopy

#### 2.3.1. Light microscopy

After fixation in formalin, pieces of tissue were embedded in paraffin and sections were cut with a microtome. Sections were stained on SuperFrost microscope slides using common Haematoxylin and Eosin (HE) staining. Pictures were taken with a light microscope (Olympus BX51) of the HE stained samples and the skin pieces.

#### 2.3.2. Transmissions electron microscopy (TEM)

Preparation of the samples were done according to Arcalis (2004) with small modifications. Samples were cut into 1 mm^3^ pieces and fixed in 2% paraformaldehyde and 2.5% glutaraldehyde in phosphate buffer (0.1 M, pH 7.4, v/v) for 2 h at room temperature. Samples were washed several times with phosphate buffer (0.1 M) and then kept in buffer over night at 4°C. The samples were post-fixed with osmium tetraoxide (1%) and potassium ferricyanide (0.8%) for 3 h at room temperature. Washing steps were performed with phosphate buffer (0.1 M). On ice, acetone series were used for dehydration. Infiltration with epoxy resin (Agar low viscosity resin kit, Agar Scientific Ltd., Essex, UK) was done in several steps for 3 h on ice. The sample was kept at 4°C in pure resin overnight. Polymerization was done in pure resin in inclusion molds at 60°C for 24 h. From the blocks, ultrathin section were cut and mounted on copper grids. Staining was done with 1% (w/v) aqueous uranyl acetate. The ready prepared sample grids were inspected with a FEI Tecnai G2 operating at 160 kV.

### 2.4. RNA extraction and sequencing

The FastRNA Pro RED KIT (MP Biomedicals, Santa Ana, CA) was used to extract total RNA out of three independent biological replicates. Fungal biomass was scratched from the skin surface to perform RNA extraction. Following the Life Technologies manual, of 1–8 μg total RNA, mRNA were poly(A)-enriched with the Dynabeads mRNA DIRECT Micro Kit (Ambion by Life Technologies, Carlsbad, CA) and the libraries were created using the Ion Total RNA-Seq kit v2 (Life Technologies, Carlsbad, CA). In all the steps quality and quantity of the RNA and in later steps cDNA was measured with the Agilent 2100 Bioanalyzer (Agilent Technologies, Santa Clara, CA) and Qbit Fluorometer 2.0 (Life Technologies, Carlsbad, CA). Targeted length of the library was selected to 290 bp with Pippin Prep instrument (Sage Science, Beverly, 7 MA). Sequencing was performed using Ion Proton Technology and the HiQ sequencing kit.

### 2.5. Bioinformatics

#### 2.5.1. Reads mapping

All reads were mapped with STAR 2.4.1d (Dobin et al., 2013) with -alignIntronMin 15 -alignIntronMax 2000 -outFilterIntronMotifs RemoveNoncanonicalUnannotated -chimSegmentMin 12 -chimJunctionOverhangMin 12 -alignSJDBoverhangMin 10 against the concatenated human and *Exophiala dermatitidis* genomes. This allowed us to assess how many reads were preferentially mapping to human. Unmapped reads were mapped against a concatenated database of rRNAs by using mapping parameters allowing a higher sensitivity –outFilterScoreMinOverLread 0 –outFilterMatchNminOverLread 0 –outFilterMatchNmin 20 The reads mapping against *Exophiala dermatitidis* were then used to find differentially expressed genes, detect chimeric RNAs and to improve the current *Exophiala dermatitidis* annotation. Count of mapped reads on annotation elements was done with featureCounts v1.4.6p2 (Liao et al., 2014). The identification of differentially expressed genes was done with the R (R Core Team, 2016) module edgeR (Robinson et al., 2010), while the functional enrichment of the significantly regulated genes was done with GoStat and Kobas, with BlastP (*E*-value < 1·10^−08^) (Falcon and Gentleman, 2007; Xie et al., 2011). Revigo (Supek et al., 2011) was used to summarize the lists of overrepresented Gene Ontology terms. Sample to sample distance was assessed with R. Fusion transcripts were detected with the STAR-Fusion pipeline: https://github.com/STAR-Fusion/STAR-Fusion while circular RNAs (circRNAs) were detected by looking directly at the read mapping patterns. In order to be reported, circular RNAs had to be supported by at least 5 split reads in two replicates of the same experimental condition. Fusion transcripts were reported if they appeared in at least three replicates of the same condition and with at least ten sustaining reads in each replicate.

#### 2.5.2. Annotation

The annotation of ncRNAs was taken from Blasi et al. (2015) while the protein coding annotation was obtained from Chen et al. (2014). Genes annotation were enriched by assembling the reads into transcripts. To this end the sequenced reads were assembled with Trinity 2.0.6 and Cufflinks 2.2.1 (Trapnell et al., 2012; Haas et al., 2013). The assembled transcripts were then combined with PASA (Haas et al., 2003). These transcripts were used to update (splice variants, UTR) and enrich the protein-coding gene annotation. Transdecoder (Haas et al., 2013) was applied to the PASA-assembled transcripts to find new putative protein-coding genes. PASA-Transcripts that were not considered coding by Transdecoder and by CPAT (Wang et al., 2013) (*p*-value < 0.01), that did not show significant (*p*-value > 0.001) sequence homology against SWISSPROT when searched with Blast (Altschul and Gish, 1996) and that did not contain Pfam-domain (Finn et al., 2014) (*p*-value > 0.001) when searched with HMMER (Rawlings et al., 2014) were classified as non-coding. The transcripts that could not be unequivocally classified as either coding or non-coding were classified as transcript with unknown functions. Samtools (Li and Durbin, 2009) were used to process the mapped reads. Annotation overlap were studied with the Bedtools (Quinlan and Hall, 2010).

Interproscan (Jones et al., 2014) as well as the CaZy-, Merops- and TCDB-databases (Saier et al., 2006; Cantarel et al., 2009; Rawlings et al., 2014) were used to functionally annotated protein coding genes.

Conserved coding and non-coding elements were detected with RNAcode (Washietl et al., 2011) (*p*-value < 0.01) and RNAz (Washietl et al., 2005b) (probability *P* > 0.9), respectively. To this aim a multiple genomes alignment of *Exophiala dermatitidis, Cladophialophora immunda, Fonsacaea pedrosi, Hortea werneckii* and *Candida albicans* was generated with the multiz pipeline (Blanchette et al., 2004).

The pipelines for the RNAseq mapping, differential expression, chimeric RNA annotation and gene functional annotation were implemented with snakemake (Köster and Rahmann, 2012).

## 3. Results

### 3.1. RNA sequencing of *exophiala dermatitidis*

The transcriptional landscape of *Exophiala dermatitidis* during infection was studied by growing *Exophiala dermatitidis* on human *ex-vivo* skin models as well as on a prewetted Nylon membrane for 1 week (see Materials and Methods). The skin infection and control experiments were sequenced in triplicates on the Ion Proton platform, yielding a total of 286 million reads with an average length of 170 nts. The reads were mapped against the concatenated human and *Exophiala dermatitidis* genomes, in order to separate the reads based on their human or fungal origin. From the 286 million reads, between 32% and 78%, depending on the sequencing run, mapped to the fungal genome, i.e., 154.5 million reads could be mapped to the *Exophiala dermatitidis*. 173,133 reads were mapped to the human genome (Supplementary Table 1 for more details). The reads mapping against the human genome were discarded from further analyses. The unassigned reads were remapped to a set of saccharomycetes and human rRNAs with STAR parameters increasing the mapping sensitivity (see Materials and Methods). Between 83% and 98.76% of the unmapped reads were assigned to rRNAs, indicating that the poly(A) enrichment protocol did not completely discard rRNAs. Despite the rRNA contamination, sample expression similarity assessed with principal component analysis fits well with the expectation from the experimental design (See Supplementary Figure 2).

### 3.2. Infection

**Figure 1:**
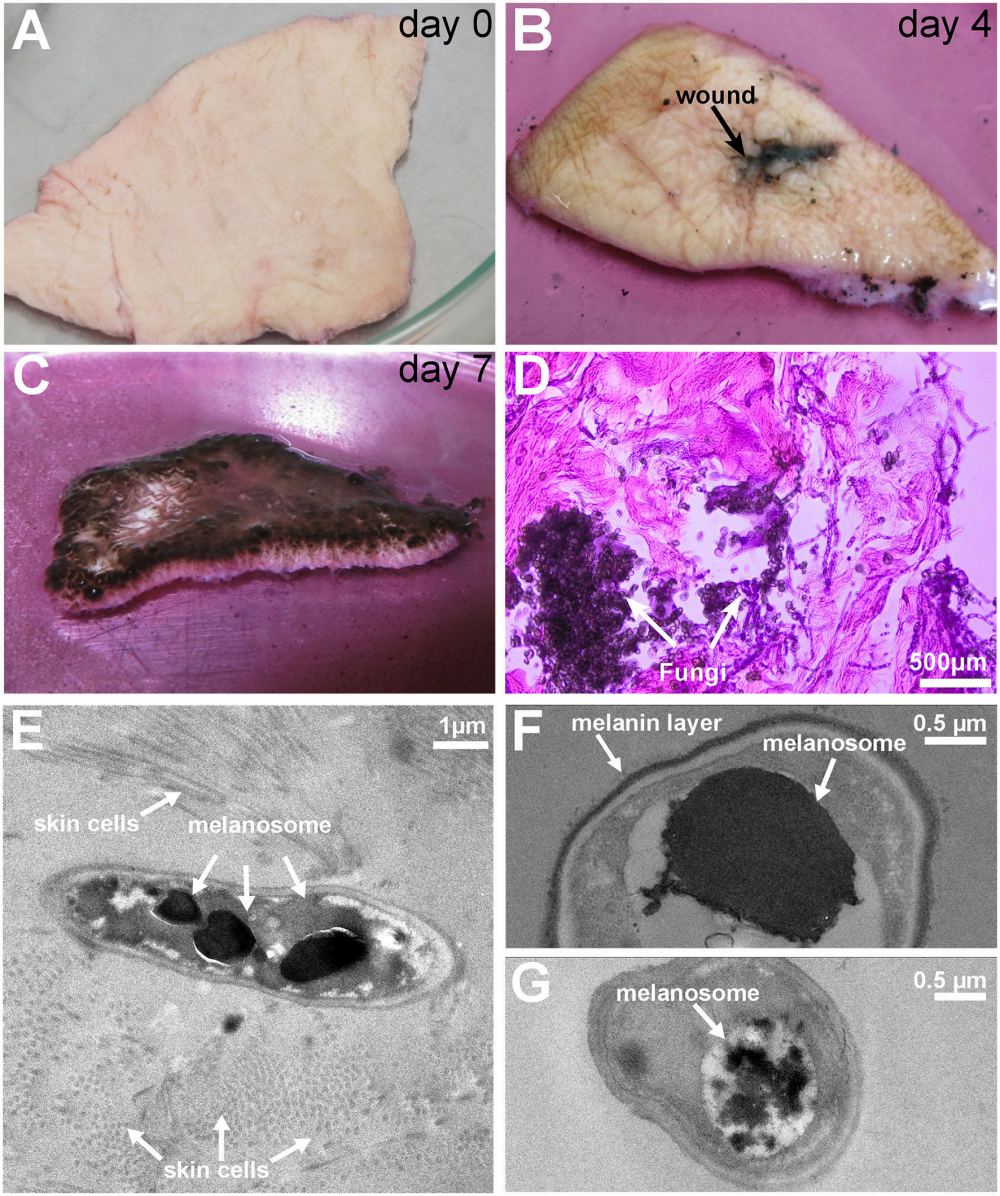
**Pictures of the colonization and invasion of *ex-vivo* skin samples by *Exophiala dermatitidis* at 37° and TEM pictures after 1 week**. **(A)** At day 0, *ex-vivo* skin samples were cut in approximately 5 × 5 cm pieces and inoculated with *Exophiala dermatitidis*. **(B)** At day 4 growth in and around the wound (black spot in the middle) could already be observed. **(C)** At the end of the experiment (day 7), the upper skin surface was completely covered by the fungus. **(D)** HE staining of the infected skin. Skin is stained in pink and the fungus is stained in deep purple. After 7 days *Exophiala dermatitidis* had penetrated deep inside the skin. **(E)** Melanosomes inside a fungal cell surrounded by skin cells. **(F)** Vacuole filled with flocculated melanin and melanin layer surrounding the cell. **(G)** Flocculated melanin in a vacuole.

The inoculated skin regions were found to be populated with *Exophiala dermatitidis* 4 days post infection. After 7 days the fungus had completely covered the skin surface (see Figures 1A–C). Further the fungus successfully invaded the skin as can be seen from the HE stained microscopic specimen shown in Figure 1D and from Supplementary Figure 2. *Exophiala dermatitidis* also started to colonize the undersurface of the skin (see Supplementary Figure 1).

The successful skin entry and growth of fungal cells were also validated by TEM pictures (see Figures 1E–G) where the incorporation of fungal cells into skin fibers can clearly be seen. The black electron dense bodies scattered in the fungal cells are evidence of melanosomes comparable to findings reported in the black yeast *Fonsacaea pedrosi* (Franzen et al., 1999, 2008). Another electron dense matter can be seen as melanin layer surrounding the cell wall. Morphological forms, especially melanosome-like electron dense spots, are heterogeneously scattered between individual cells (see Figures 1E–G) which might be due to differences in contact between skin and individual fungal cells.

The observed increased melanin production is in line with the strong upregulation of Tyrosine aminotransferase (16-fold) and Hydroxyphenylpyruvate dioxygenase (4-fold), two enzymes involved in the production of melanin through the L-Tyrosine degradation pathway (see **Figure 4D**).

### 3.3. Genome re-annotation

#### 3.3.1. Conserved elements

The *Exophiala dermatitidis, Cladophialophora immunda, Fonsacaea pedrosi, Hortea werneckii* and *Candida albicans* genomes were aligned with multiz (Blanchette et al., 2004). RNAz (Washietl et al., 2005a) and RNAcode (Washietl et al., 2011) were run on it in order to detect non-coding and coding elements, respectively. A total of 11168 conserved regions covering 58% of the genome were returned by the alignment pipeline. RNAz detected a total of 895 conserved non-coding loci with a *P*-score>0.9. 683 hits overlapped 681 protein coding genes among (348 UTRs, 711 CDS). 182 elements overlapped with non-coding transcripts, 30 with annotated non-coding RNAs and 89 hits were intergenic. Because RNAz hits have no strand information a single hit might overlap to transcript located on both strands. RNAcode predicted 24,613 conserved coding elements with a *p*-value < 10^−3^. Among them 23,200 (94.2%) mapped to annotated proteins, 312 mapped to non-coding transcripts and 150 mapped to the list of transcripts that could not be strictly classified as coding or non-coding.

Three unstructured but conserved snoRNAs, i.e., snR72, snR74, and snR4 were found by specifically looking at conserved intronic regions in *Exophiala dermatitidis* and subsequently by searching for homologous sequences in fungi with RNAlien (Eggenhofer et al., 2016) (see **Figures 3A,B**).

#### 3.3.2. RNAseq-based re-annotation

**Figure 2:**
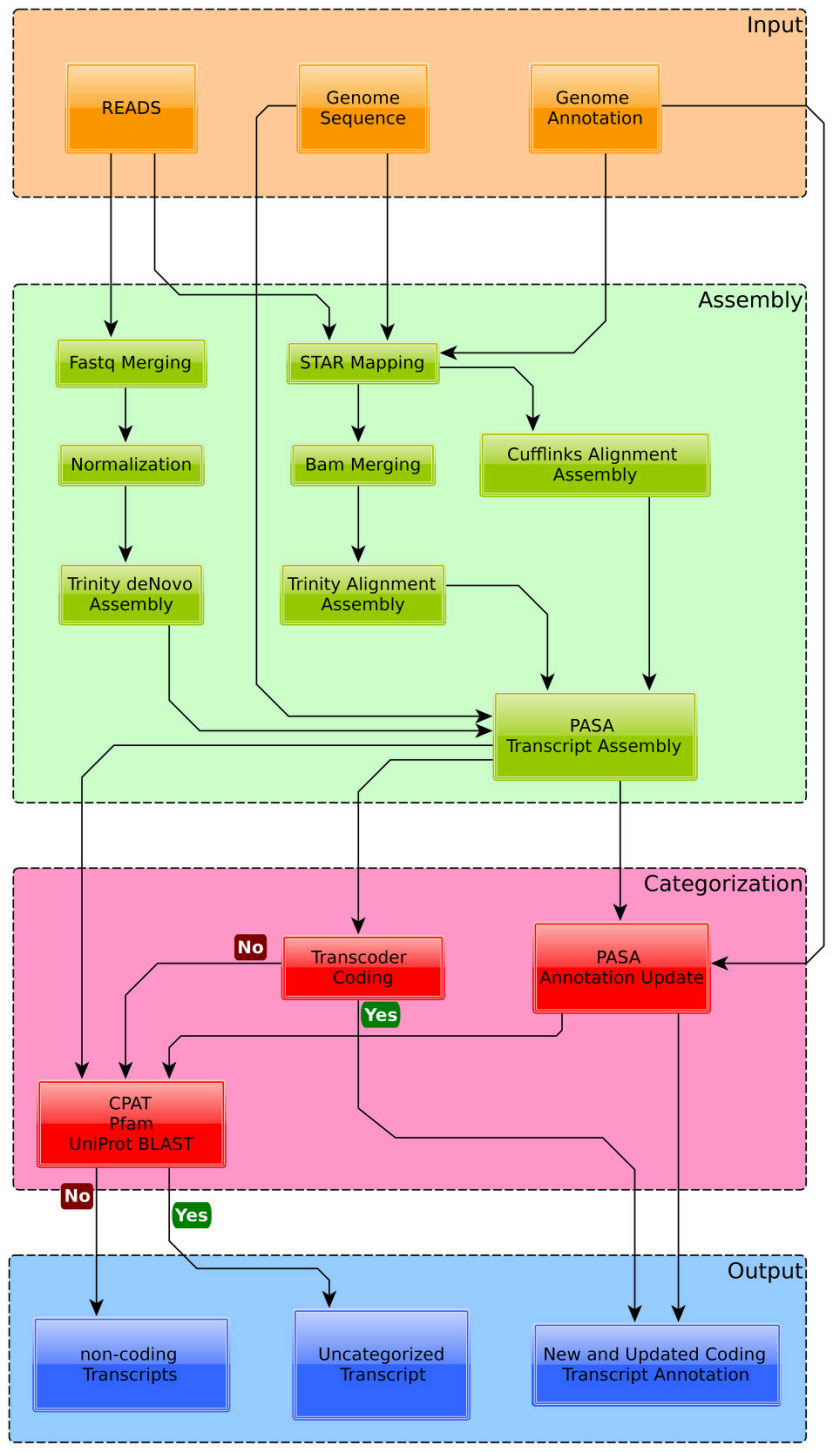
**Overview of the annotation pipeline used to augment the current genome annotation of *Exophiala dermatitidis***. Transcripts were assembled with Trinity and Cufflinks and mapped back to the genome with PASA. Transcripts overlapping with *Exophiala dermatitidis* coding regions were used to find new splice variants and (re-)annotate UTRs. Transcripts that were considered coding by Transdecoder were classified as new proteins. The other transcripts were tested for their coding potential with CPAT, Pfam and by blasting them against Uniprot in order to differentiate between truly non-coding transcripts and non-coding transcripts with coding potential.

The current transcript annotation of *Exophiala dermatitidis* was updated by using RNA-Seq data from the skin and skin-control experiments (See Methods and Figure 2 for more details).

Out of the 9577 protein coding transcripts annotated in *Exophiala dermatitidis*, 3391 were not modified by PASA while 4168 new splice variants were detected (see Supplementary Table 1). In 18 cases two neighboring transcripts were merged into one (see Supplementary Table 1). Further a total of 2284 coding transcripts, mapping to 1241 loci and without overlap with known CDS, were found. Among them 555, mapping to 348 loci, had no UTR overlap with the official genome annotation (see Supplementary Table 1).

Given the genome size of *Exophiala dermatitidis* the total number of 10907 protein-coding loci is in-line with that of *Exophiala mesophila* (29Mb,9121loci), *Exophiala sideris* (29Mb,10114 loci) and *Exophiala spinifera* (32MB, 12049 loci). 684 gene loci overlapped with genomic regions contained in the multiple genomes alignment. The 2284 predicted protein-coding transcripts were blasted against the nr-database. 578 proteins from 131 loci had a hit with a *E*-value < 0.001. 92 out of the 131 loci overlapped with regions of the multiple genomes alignment. 387 proteins from 133 loci could be functionally annotated (see Methods).

**Figure 3:**
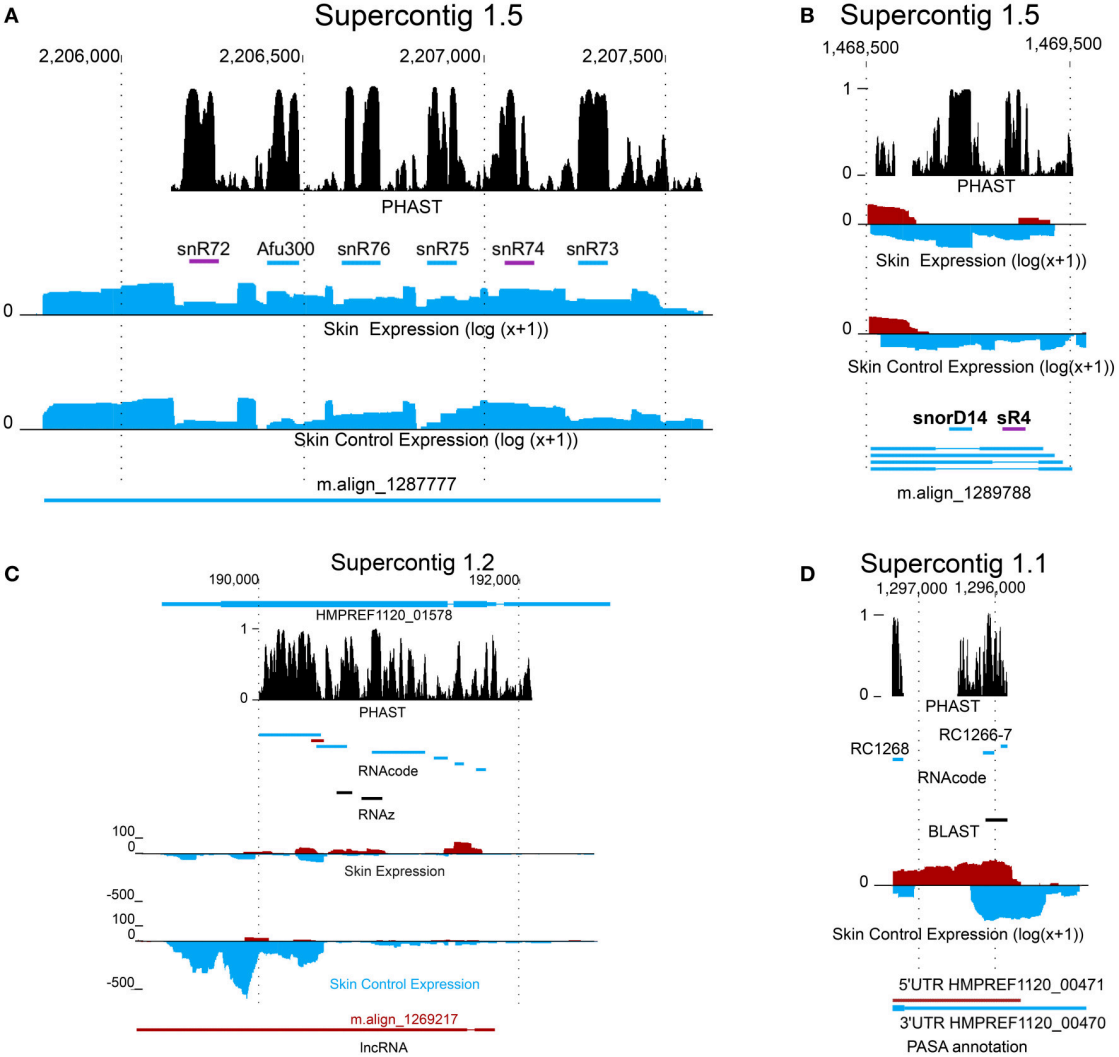
** New coding and non-coding genes in *Exophiala dermatitidis***. **(A)** m.align_1287777 is a spliced non-coding transcript containing intronic snoRNAs. snR72 and snR74, both shown as were annotated in this study. **(B)** The transcripts encoded in region Supercontig_1.5 1468500-1469500 contains two intronic snoRNAs, snorD14 and sR4. snorD14 is upregulated when *Exophiala dermatitidis* grows on the *ex-vivo* skin samples. **(C)** lncRNA m.align_1269217 and HMPREF1120_01578 are antisense and exhibit opposite regulation pattern. They are located in a conserved region where the secondary structure and the coding potentials are constrained. **(D)** An homolog of *Candida albicans* metallothionein, shown as black bar, was found in a conserved region overlapping with the 3UTR of HMPREF1120_00470. The coding potential of the region encoding the metallothionein is further sustained by the RNAcode predictions RC1266 and RC1267.

Further we looked at the presence of new non-coding RNAs by fetching all PASA assembled transcripts that did not overlap with protein-coding loci, that did not contain pfam-domain, that were not reported as coding by CPAT (Wang et al., 2013) (coding *p*-value >0.001) and that did not show sequence homology to SwissProt (Blastp *E*-value >0.001). A total of 7778 non-coding transcripts mapping to 5017 gene loci were found. Four snoRNAs, homologous to snR4, snorD14, snosnR55, and snosnR61 are located in introns of long non-coding transcripts (see Figure 3B). Each snoRNA is flanked by canonical splice sequences (GT/AG).

The transcripts that were neither classified as coding by transdecoder nor rejected as non-coding by CPAT, Pfam or Blast (see Methods) were classified as transcript of ambiguous type (TAT). A total of 6630 TATs from 5800 loci were found. Among these 5800 loci, 3133 overlapped with RNAcode predictions. While no information on the TAT function is available, one TAT, align_m.1287777 is hosting a snoRNA cluster containing snR72, snR74, snR75, snR76, snR77, Afu300 (see Figure 3A). SnoRNA clusters have been detected in a multitude of different eukaryotic organisms, including *Saccharomyces cerevisiae*, which may suggest an ancient origin of this gene arrangement (Liang et al., 2002). Similar to the snR4-snorD14 cluster, each snoRNA is flanked by canonical splice sites indicating that they are actively spliced out of the TAT transcript.

#### 3.3.3. Circular and chimeric RNA

The occurrence of circular RNAs and fusion transcripts in the transcriptome data was investigated. CircRNAs were searched by examining reads that contained apparent splice junctions connecting the end (start) of a split read fragment to the start (end) of a downstream (upstream) fragment. Five circularized transcripts were found in at least two replicates of the skin with at least 6 supporting read and 4 were found in the skin control.

The presence of fused transcripts in *Exophiala dermatitidis*, i.e., distant transcripts connected by split reads were recently reported in Blasi et al. (2015). In this study, 126 fusion transcripts supported by at least 10 reads in all replicates of a given condition were found. Sixty two of the fusion transcripts were specific to the control condition, 43 were found exclusively in the skin experiment and 21 were found in both conditions.

### 3.4. Transcript expression

Functional enrichment of the 100 most highly expressed coding genes in the skin and control samples was analyzed. In the control samples, genes with domains related to Band 7 proteins were enriched (HMPREF1120_07454, HMPREF1120_01950, HMPREF1120_06159) (fdr = 0.001) and Histone-related proteins (HMPREF1120_06310, HMPREF1120_01816, HMPREF1120_06252). The 100 most highly expressed proteins in the skin experiment were related to Heat shock protein 70 (Hsp70) (HMPREF1120_02626, HMPREF1120_07756, HMPREF1120_08142,fdr = 0.002; 3/5 genes), phosphorylcholine metabolism (HMPREF1120_09233, HMPREF1120_04356 fdr = 0.004, 2/2) and translation elongation (HMPREF1120_08281, HMPREF1120_00844, fdr = 0.004, 2/2). Hsp70 is known for having function in protection and repair of cells after stress. In addition, in *Candida albicans* two members of the Hsp70 family are expressed on the cell surface and function as receptors for antimicrobial peptides (López-Ribot et al., 1996; Sun et al., 2010). In the pathogenic fungus *Paracoccidioides brasiliensis* Hsp70 was also found to be induced in the mycelial to yeast transition (da Silva et al., 1999). Phosphorylcholine (PC) locates on the cell surface of various pathogenic prokaryotes and eukaryotes. While PC is targeted by the host immune system, PC can also modulate the response of the immune system, allowing the pathogen to remain undetected. Through modifications of the PC, the pathogenic microbes and bacteria can hide from or modulate the immune response (Clark et al., 2012; Clark and Weiser, 2013).

### 3.5. Differential expression

#### 3.5.1. ncRNAs

It was checked if ncRNAs as annotated in Blasi et al. (2015) were differentially expressed. While no non-coding RNA was significantly downregulated in the skin experiment, 4 ncRNAs (SRP, Afu_300, snorD14,U6) were upregulated with a fdr < 0.05 (see Supplementary Table 1). snorD14 (U14/snR128), which is upregulated 10 times (corrected fdr = 0.026) in the skin experiment vs. control, is a C/D Box snoRNA that is found in plants, fungi and animals. Genetic depletion and mutation of snorD14 in yeast showed that it is required for growth and that loss of snorD14 disrupts pre-rRNA processing (Samarsky et al., 1996). Interestingly snorD14 was shown to be contained in the extracellular vesicles (EV) of *Saccharomyces cerevisiae* and of human fungal pathogens (*Cryptococcus neoformans, Paracoccidioides brasiliensis* and *Candida albicans*) (Peres da Silva et al., 2015). Similarly we could identify this snoRNA in the extracellular vesicles of *Exophiala dermatitidis* and *Cladophialophora immunda* growing at 37°C (manuscript in preparation).

Like snorD14, SRP-RNA (7SL) was also found in EV of *Exophiala dermatitidis* and was strongly upregulated in the skin experiment. With the exception of Srp74 that was significantly upregulated (fdr = 0.006), the other SRP components annotated in *Exophiala dermatitidis* were not differentially expressed.

Sixty four putative lncRNAs were significantly (fdr ≤ 0.05) upregulated (see Supplementary Table 1). We looked at the set of significantly regulated proteins overlapping with the group of significantly regulated lncRNAs. We found that 7 protein-coding loci were significantly upregulated (HMPREF1120_06710, HMPREF1120_00114, HMPREF1120_01575, HMPREF1120_04063, HMPREF1120_ 01423, HMPREF1120_01437, HMPREF1120_01107), and 4 were significantly downregulated (HMPREF1120_01578, HMPREF1120_06121, HMPREF1120_00784, HMPREF1120_04399) (see Figure 3C).

The region covered by HMPREF1120_01578 is interesting as it contains signals for coding and non-coding elements. There is a long non-coding RNA antisense to the coding locus. The genomic region is well conserved as can be seen from the PHAST score in Figure 3C. RNAcode tells us that both strands have significant coding potential, i.e., that the antisense lncRNA might also have at least one conserved ORF. Finally we find RNAz hits that overlap with one of the RNAcode predictions, the non-coding RNA and the coding locus. These annotations indicate that the protein and/or lncRNA loci encode transcripts that might be multi-functional, working either as a coding elements and/or as ncRNAs.

Fourteen putative TAT were significantly upregulated and 5 were downregulated. Two upregulated TAT overlapped with two upregulated protein coding genes.

#### 3.5.2. Functional enrichment of regulated coding genes

Over 2000 protein coding genes were differentially expressed (DEG) with an fdr < 0.05 and |log_2_*FC*| > 1 between the skin and control experiments (see Supplementary Table 1). Even at a more stringent threshold on the fold change (|*log*_2_*FC*| > 2), 1016 DEG were found (490 upregulated and 526 downregulated) with an fdr < 0.05 between the skin and control experiments (see Supplementary Table 1).

#### 3.5.3. Upregulated genes

The largest enriched functional category in the set of upregulated genes was DNA replication (fdr = 3.3·10^−4^). Together with the categories DNA metabolic process (fdr = 5.0·10^−3^) and rRNA metabolic process (fdr = 7.0·10^−3^), it indicates that *Exophiala dermatitidis* seems to be in a replicative state when growing on skin. The enrichment analysis of protein domains in the upregulated genes is in line with the GO analysis. P-loop containing nucleotide triphosphate hydrolase (NTPase) (G3DSA:3.40.50.300, fdr = 4.9·10^−15^, 101 genes; IPR027417, fdr = 1.67·10^−14^, 104 genes; SSF52540, fdr = 4.56·10^−14^; 104 genes) is the most significantly enriched term. In *Toxoplasma gondii* NTPase are essential for the parasite proliferation (Nakaar et al., 1999). Helicase C (Pfam00271, fdr = 1.25·10^−8^, 32 genes), helicase_CTER (PS51194, fdr = 5.66·10^−8^, 32 genes), DEAD-like helicase superfamily (SM00487, fdr = 5.57·10^−6^, 19 genes), Helicc (SM0049, fdr = 5.57·10^−6^, 19 genes), helicase ATP binding (PS51192, fdr = 4.67·10^−8^, 32 genes), helicase superfamily 1/2 (IPR014001, fdr = 7.97·10^−9^, 32 genes) and helicase C terminal (IPR001650, fdr = 7.97·10^−9^, 32 genes) are enriched and correspond to the overrepresented GO terms related to DNA replication. RNA helicases and especially DEAD box RNA helicases are important for RNA synthesis and also function as pre-mRNA processing and in ribosome biogenesis (Franzen et al., 2008). Mini chromosome maintenance complex (PS50051, fdr = 0.0157, 5 genes) is important in the initiation and elongation phases in DNA replication. AAA+ ATPase domain (IPR003593, fdr = 0.0002, 29 genes) is important for membrane fusion, proteolysis and DNA replication (Ogura and Wilkinson, 2001). SNF2-related, N-terminal domain (IPR000330, fdr = 0.0090, 11 genes) is related to transcription regulation, DNA repair, DNA recombination and chromating unwinding.

In Merops two families of proteases are overrepresented, C26 (fdr = 0.0010, 13 genes) and S16 (fdr = 0.0043, 6 genes). C26 is a gamma-glutamyl hydrolase which is either secreted or located in lysosome while S16 Lon protease is a known bacterial and fungal virulence factor (Takaya et al., 2003; Breidenstein et al., 2012; Cui et al., 2015).

**Figure 4:**
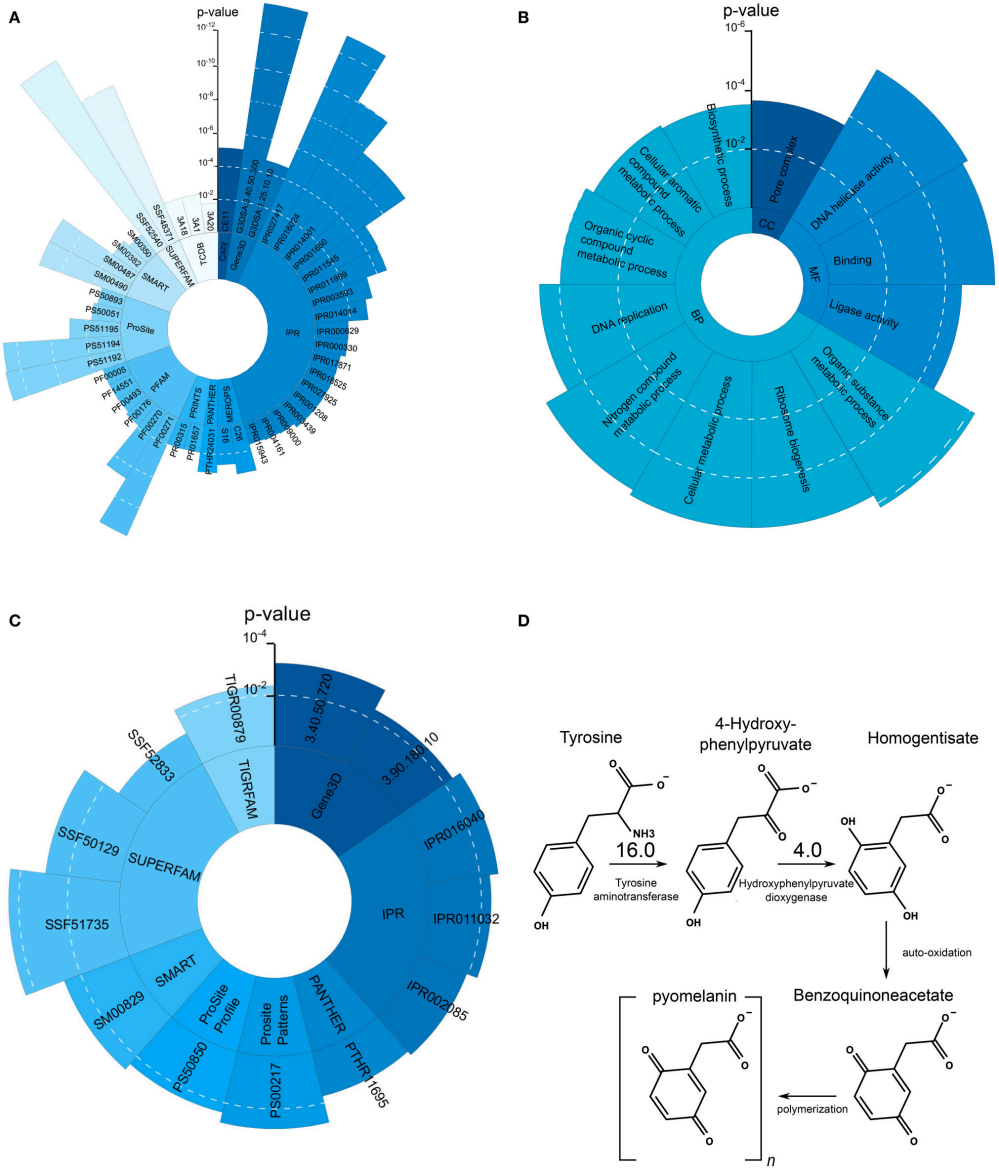
**Significantly overrepresented protein domains and GO terms as well as L-Tyrosine melanin metabolism**. **(A)** Overrepresented protein domains in the set of upregulated genes during growth on skin. Each blue shade represent another enriched domain category. **(B)** Overrepresented GO terms in the set of upregulated genes during growth on skin. **(C)** Overrepresented GO terms in the set of downregulated genes during growth on skin. **(D)** L-Tyrosine degradation pathway leading to the production of pyomelanin. Compared to the control condition Tat and HppD are upregulated 16 and 4 times, respectively.

In TCDB the Peroxisomal Protein Importer (PPI) Family (3A20, fdr = 0.016, 8 genes), the ATP-binding Cassette (ABC) Superfamily (3A1, fdr = 0.0038, 15 genes) and the nuclear mRNA Exported (mRNA-E) Family (3A18, fdr = 0.0038, 17 genes) were enriched in the set of upregulated proteins. An overview of the most enriched protein domains and GO terms can be found in Figures 4A,B.

#### 3.5.4. Downregulated genes

Oxidoreductase activity and carbohydrate transport were the most significantly enriched term in the set of downregulated genes. 39 protein domain annotations were enriched (see Supplementary Table 1). NAD(P)-binding domain (G3DSA:3.40.50.720, fdr = 0.0043, 24 genes; G3DSA:3.90.180, fdr = 0.0422, 7 genes; IPR016040, fdr = 0.0019, 61 genes, IPR011032, fdr = 0.0042, 18 genes; PTHR11695, fdr = 0.0502, 7 genes; SSF51735, fdr = 5.2191·10^−5^, 62 genes) is contained in many different dehydrogenases and is related to the utilization of nitrogen source (Gough et al., 2001) in yeast. IPR005828 and IPR005829 are domains involved in the binding and transport of various carbohydrates and particularly sugars (IPR005829, fdr = 0.082, 14 genes; PF00083, fdr = 0.086, 16 genes; PS00217, fdr = 0.0015, 13 genes; TIGR00879, fdr = 0.0046, 12 genes).

KEGG pathways related to metabolism of xenobiotics by cytochrome P450 (ko00980, fdr = 0.0317, 3 genes), Glycosphingolipid biosynthesis (ko00603, fdr = 0.0317, 2 genes) and N-glycan biosynthesis (ko00513, fdr = 0.0317, 2 genes) are overrepresented. An overview of the enriched protein domains can be found in Figure 4C.

### 3.6. Nutrient acquistion

#### 3.6.1. Carbon

**Figure 5:**
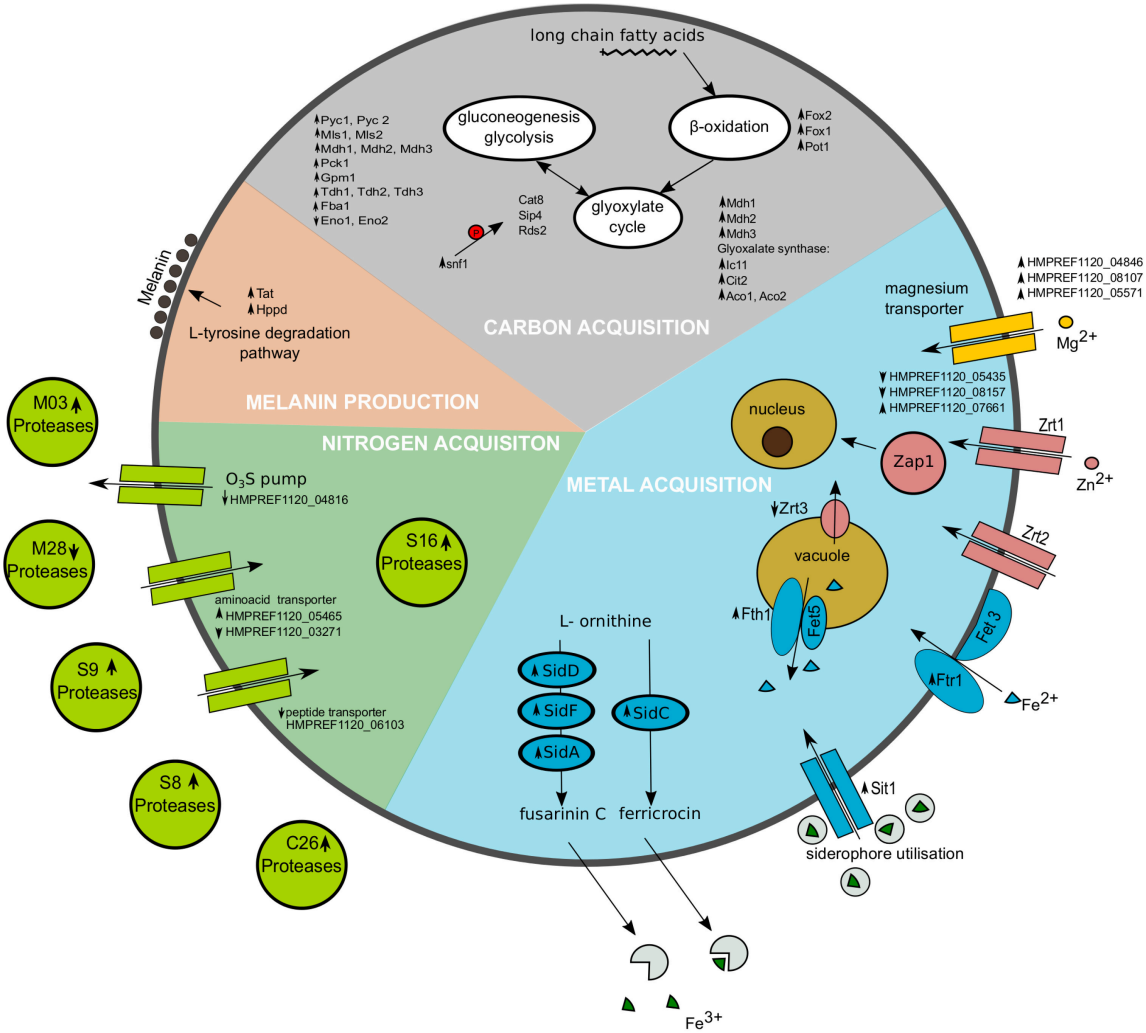
**Representation of selected cellular processes significantly regulated in *Exophiala dermatitidis* when growing on skin**. Metal acquisition is shown in the blue region, Nitrogen acquisition in the green region and carbon acquisition in the gray region. Melanin production is found in the salmon pink region. Upregulated genes are marked with upward pointing arrows, while downregulated genes are marked with downward pointing arrows. The red P in the carbon acquisition part indicates phosphorylation.

In yeast, Snf1 kinase is activated under gluconeogenic conditions leading to the activation of gluconeogenesis and glyoxylate cycle. Snf1 in *Exophiala dermatitidis* is strongly upregulated (fdr = 0.003; 5.46) (see Supplementary Table 1, Figure 5). Since most of the transcriptional regulators triggering gluconeogenesis in *Saccharomyces cerevisiae* are also available in *Candida albicans* and *Exophiala dermatitidis*, similar regulatory mechanisms might exist in these fungi (Turcotte et al., 2010; Fleck et al., 2011). In fact, in the skin environment both the gluconeogenesis pathway and the glyoxylate cycle of *Exophiala dermatitidis* is regulated in a way similar to what is seen in *Candida albicans* upon phagocytosis (Lorenz et al., 2004) (see Supplementary Table 1, Figure 5). The acetyl-CoA needed by the glyoxylate cycle is probably obtained from the β-oxidation pathway as all of its enzymes are significantly upregulated in the skin condition in *Exophiala dermatitidis* (see Supplementary Table 1). This is similar to what is seen in *Candida albicans* (Fleck et al., 2011).

#### 3.6.2. Nitrogen

Homologs of *Candida albicans* aspartyl proteases, which are associated with the dissociation of amino acids from the host (Fleck et al., 2011), were found in *Exophiala dermatitidis* but none of them were upregulated.

In *Onygena corvina* a mix of three protease families, S8, M28, and M3 was shown to be sufficient to break down keratin (Huang et al., 2015). Nine S8 proteases were found in *Exophiala dermatitidis*. Only HMPREF1120_07613 and HMPREF1120_06376 were significantly regulated in skin w.r.t. control by a factor of 2.56 and 0.22, respectively. Six genes are members of the M28 protease family but only HMPREF1120_00135 is significantly regulated (fdr = 0.0027; 0.015). Two M03 proteases, HMPREF1120_07166 and HMPREF1120_06271, were found in *Exophiala dermatitidis* but only the former was significantly regulated (fdr = 0.0077; 3.18).

HMPREF1120_07218, a prolyl oligopeptidase, is the second most significantly upregulated protease (fdr = 0.0029; 4.41), and was shown to be a virulence factor in the bacterium *Porphyromonas gingivalis* (Nelson et al., 2003) and might be connected to virulence in the dermatophytes *Trychomyces rubrum* and *Trychophyton mentagrophytes* (Kaufman et al., 2005).

In *Exophiala dermatitidis* 12 proteins are classified as amino acid transporters. HMPREF1120_05465 is upregulated 4.86 times during skin infection, HMPREF1120_03271 is downregulated 3.81 times in skin, while the other genes are not significantly regulated. Seven oligopeptide transporters are found in *Exophiala dermatitidis* among which three are related to *Saccharomyces cerevisiae* OPT (oligopeptide transporter) family. HMPREF1120_06103 is the only significantly regulated peptide transporter (fdr = 0.03; 0.31).

#### 3.6.3. Metal

In *Exophiala dermatitidis* during skin infection, three out of 13 annotated magnesium transporter, i.e., HMPREF1120_04846, HMPREF1120_08107 and HMPREF1120_05571, are significantly upregulated by a factor 6.53, 2.87, and 3.05, respectively.

During skin infection, the *Exophiala dermatitidis* Zap1 homolog (HMPREF1120_08244; Blast *E*-value 4·10^−25^) is significantly upregulated (fdr = 0.01; 2.56). *Exophiala dermatitidis* contains 7 zinc transporters homolog to Zrt1 and Zrt2. Two of them are downregulated (HMPREF1120_05435, HMPREF1120_08157) while one is upregulated (HMPREF1120_07661) when the fungus is growing on skin. Zrc1 and Cot1, two proteins involved in zinc internalization (Conklin et al., 1992; Zhao and Eide, 1997) are not significantly regulated, while Zrt3, a protein associated with zinc mobilization (MacDiarmid et al., 2000), is downregulated (fdr = 0.044; 0.43).

In *Exophiala dermatitidis*, no copper transporter and no metallothionein, are annotated in the genome. Still the region located in Supercontig_1 between 1295848 and 1296128 was found to be significantly (tBlastn *E*-value < 10^−19^) similar to Cdr2 (see Figure 3), a metallothionein (Ding et al., 2014). This is a conserved region with significant RNAcode signals overlapping with the 3'UTR region of HMPREF1120_00470. Other genes involved in Cu uptake, like Crt2 (HMPREF1120_07198), Crt3 (HMPREF1120_00028) Fre (HMPREF1120_00949), Ccc2 (HMPREF1120_05014), Atx1 (HMPREF1120_03801) and Sur7 (HMPREF1120_00735) (Ding et al., 2014) were found in the genome, but were not significantly regulated in the skin experiment.

Genes related to the reductive Iron uptake pathway, like Fres (HMPREF1120_ 00949), Ccc1 (Li et al., 2001) (HMPREF1120_04139, HMPREF1120_03992), or the gene pairs Fth1/Fet5 and Ftr1/Fet3 (HMPREF1120_01589, HMPREF1120_ 01590; HMPREF1120_02753, HMPREF1120_02754; HMPREF1120_04509, HMPREF1120_04510) were found in *Exophiala dermatitidis*. Still only HMPREF1120_ 04509 (putative Ftr1 or Fth1) is significantly regulated in the skin experiment (fdr = 0.025; 6.76).

While we could not find homologs to *Candida albicans* heme- or ferritin-transporters, 8 genes homolog to Sit1, a siderophore protein responsible for iron uptake in *Candida albicans, Saccharomyces cerevisiae* and *Cryptococcus neoformans*, were found in *Exophiala dermatitidis* (Ding et al., 2014). Three of them (HMPREF1120_07838, HMPREF1120_02555, HMPREF1120_01434) were significantly regulated (fdr = 0.006, 0.011, 0.031; 14.26, 7.07, 4.473) in skin w.r.t. control. Further the enzymes responsible for the synthesis of the siderophores fusarinin C and ferricrocin, i.e., SidD, SidF, SidA and SidC (HMPREF1120_01440, HMPREF1120_01438, HMPREF1120_07635, HMPREF1120_07636) (Chen et al., 2014) were upregulated when *Exophiala dermatitidis* grew on skin (fdr = 0.012, 0.010, 0.0334, 0.0028; 8.74, 4.55, 2.45, 7.31).

### 3.7. Virulence related genes

We checked for differential expression of known virulence factors of *Exophiala dermatitidis, Blastomyces dermatitidis, Candida albicans, Aspergillus fumigatus* and *Cryptococcus neoformans* found in the PHI database (Winnenburg et al., 2008). Three genes related to virulence were found to be upregulated: 60S ribosome biogenesis protein Brx1 (PHI:2546, Q4WKJ9, HMPREF1120_02997, fdr = 0.0061, logFC = 2.18), ATP dependent RNA helicase mak5 (PHI:2549, Q4WMS3, HMPREF1120_06261, fdr = 0.0063, logFC = 2.46) and nuclear export protein Noc3 (PHI: 2551, Q4WZG4, HMPREF1120_05871, fdr = 0.0069, logFC = 2.39).

#### 3.7.1. Dimorphism

*Exophiala dermatitidis*, like *Candida albicans* (Schaller et al., 1999; Albrecht et al., 2006), is a polymorphic fungus that can adopt yeast-like form or the hyphal form. Both morphologies were found in various cases of Phaehyphomycosis caused by *Exophiala dermatitidis* (Woollons et al., 1996; Myoken et al., 2003; Park et al., 2011). In the HE stained specimen it can be observed that the majority of the fungal cells are in yeast form (see Figure 1D). We searched for homologs of eight dimorphism related genes reported in Mayer et al. (2013) (see Table 1). Hgc1, a gene necessary for the formation of hyphae, showed a significant regulation (fdr = 0.012, logFC = 3.13). Further the expression of HMPREF1120_05541, a gene shown to be essential for the yeast-hyphal switch (Ye and Szaniszlo, 2000) and homolog to *Candida albicans* Efg1, is increased by a factor 7 during skin infection, indicating that a part of the fungal population growing on skin is switching to the hyphal form.

**Table 1:** **List of fungal virulence factors found in *Exophiala dermatitidis***.

**Function**	**Species**	**Gene**	**Homolog**	**Type of homology**	**DEG (fdr;fold) Rank**	**References**
Shape	*Candida albicans*	Hgc1	HMPREF1120_04169	B:4·10^−12^,PD:6·10^−35^	0.012; 3.13	Mayer et al., 2013
	*Exophiala dermatitidis*	Efg1 StuA	HMPREF1120_05541	B:4·10^−168^	0.0037; 7.01	Ye and Szaniszlo, 2000
Adherence	*Aspergillus fumigatus*	AfCalAp	HMPREF1120_05161	B:1·10^−23^,PD:1.5·10^−4^		Upadhyay et al., 2009
			HMPREF1120_03984	B:7·10^−13^,PD:5.1·10^−9^		
			HMPREF1120_05769	PD:2.22·10^−48^		
	*Candida albicans*	Ecm33	HMPREF1120_03851	B:6·10^−49^		Martinez-Lopez et al., 2006
	*Candida albicans*	Phr1	HMPREF1120_03477	B:7·10^−177^		Calderon et al., 2010
			HMPREF1120_07283	B:1·10^−85^	85	
			HMPREF1120_01763	B:4·10^−84^		
			HMPREF1120_01682	B:2·10^−66^		
	*Microsporum canis*	Sub3	HMPREF1120_08439	B:2·10^−75^		Baldo et al., 2010
Protease	*Candida albicans*	Sap1-10	HMPREF1120_00212	PD:8·10^−93^ B:8·10^−19^		Mayer et al., 2013
			HMPREF1120_00818	PD:3·10^−77^ B:3·10^−16^		
			HMPREF1120_01981	PD:5·10^−6^		
			HMPREF1120_03062	PD:8·10^−19^		
			HMPREF1120_03766	PD:2·10^−81^ B:3·10^−26^		
			HMPREF1120_05067	PD:3·10^−22^		
			HMPREF1120_05119	PD:9·10^−90^ B:7·10^−61^		
			HMPREF1120_06343	PD:3·10^−115^ B:4·10^−33^		
			HMPREF1120_06360	PD:7·10^−77^ B:9·10^−46^		
			HMPREF1120_06819	PD:9·10^−74^ B:2·10^−14^		
			HMPREF1120_07351	PD:6·10^−36^		
			HMPREF1120_08062	PD:7·10^−76^ B:1·10^−46^		
			asmbl_11606|m.22873	PD:8·10^−25^		
	*Trychomyces rubrum*	Ssu1	HMPREF1120_04816	B:5·10^−115^	0.046; 0.40	Léchenne et al., 2007
Invasion	*Cryptococcus neoformans*	Plb1	HMPREF1120_00190	B:6·10^−100^		Noverr et al., 2003
			HMPREF1120_06300	B:1·10^−61^		
	*Candida albicans*	Ssa1 (Hsp70)	HMPREF1120_01564	B:0	0.0043;6.19	Sun et al., 2010
			HMPREF1120_02459	B:4·10^−30^	0.025;3.24	
			HMPREF1120_02626	B:0	7	
			HMPREF1120_04200	B:1·10^−61^	0.003;14.02	
			HMPREF1120_07756	B:0	0.004;30.06/28	
			HMPREF1120_08142	B:0	0.016;4.02/81	
			HMPREF1120_09114	B:3·10^−80^		
	*Exophiala dermatitidis*	StuA	See above			
Biofilm	*Aspergillus fumigatus*	Laea	HMPREF1120_06677	B:1·10^−72^		Fanning et al., 2012
			HMPREF1120_03377	B:1·10^−57^		
			HMPREF1120_07722	B:2·10^−57^		
			HMPREF1120_08797	B:3·10^−55^		
			HMPREF1120_02912	B:2·10^−51^		
			HMPREF1120_08429	B:3·10^−49^		
			HMPREF1120_01429	B:3·10^−43^		
			HMPREF1120_08930	B:1·10^−40^		
			HMPREF1120_05890	B:2·10^−37^		
			HMPREF1120_05291	B:5·10^−32^		
			HMPREF1120_02485	B:8·10^−24^		
	*Candida albicans*	Efg1	HMPREF1120_05541	B:6·10^−57^	9.31·10^−5^; 7.01	Dieterich et al., 2002
	*Candida albicans*	Bgl2	HMPREF1120_04141	B:7·10^−51^		Taff et al., 2012
	*Candida albicans*	Phr1	See above			
	*Candida albicans*	Exg1	HMPREF1120_04506	B:1·10^−109^		Taff et al., 2012
			HMPREF1120_06180	B:2·10^−62^		

Genes with at least one homolog in Exophiala dermatitidis are reported. Each entry contains the function, the original organism, the gene name, the corresponding homolog in Exophiala dermatitidis, the homology type (B, Blast E-value; PD, protein domain SUPERFAMILY E-value), the regulation factor, in case it is significant, and/or the expression level of the gene if it belongs to the top 100 expressed genes in the skin experiment and a reference.

#### 3.7.2. Signaling pathways

HMPREF1120_02538 (MAP kinase) and HMPREF1120_04310 (Map kinase kinase) are two members of the HOG signaling pathway and are significantly regulated (fdr 0.0031, 0.015; 5.45, 3.23) in the skin experiment. In pathogenic fungi the HOG pathway is a major controller of cellular responses to diverse external stimuli (Lenardon et al., 2010). In the Ca^2+^/calcineurin signaling pathway, the voltage-gated high-affinity calcium channel is upregulated (HMPREF1120_08350) during growth on skin (fdr 0.0086, 5.426). Results from Chen et al. (2014) show that the gene is upregulated upon acidic pH stress, which is in line with the skin having a surface pH of around 5. Interestingly two calcium transporting ATP-ase, i.e., HMPREF1120_07859 and HMPREF1120_00316, which are responsible for the transport of Ca^2+^ outside the cell, are upregulated during fungal growth on skin.

#### 3.7.3. Adherence

We searched for the 45 adhesins from six human pathogenic fungi reported by de Groot et al. (2013). Four of them, CalA from *Aspergillus fumigatus* and Ecm33, Car10, Phr1 from *Candida albicans*, were found in *Exophiala dermatitidis* (see Table 1). While none of those genes were differentially expressed, Phr1, which promotes binding to laminin and murine lung cells (Upadhyay et al., 2009) ranked among the 100 most expressed genes in the skin experiment.

Among all genes involved in biofilm formation in *Candida albicans*, only Phr1 showed a significant regulation (see Table 1). It was also ranked among the 100 most expressed genes in the skin experiment.

#### 3.7.4. Invasion

We looked for genes responsible for endocytosis or active penetration. In *Exophiala dermatitidis* 7 homologs of Ssa1, a gene involved in endocytosis in *Candida albicans* (Phan et al., 2007; Sun et al., 2010), and 3 homologs of Plb1, a gene that gives rise to endocytosis in *Cryptococcus neoformans* (Noverr et al., 2003), were found in the genome. While Plb1-homologs were neither highly expressed nor differentially regulated, 5 Ssa1 homologs were significantly upregulated and three of them belonged to the set of 100 most highly expressed genes (see Table 1).

#### 3.7.5. Secondary metabolites

SMASH (Weber et al., 2015) and SMURF (Khaldi et al., 2010) were used to find secondary metabolite biosynthesis gene clusters. SMASH returned a total of 4 NRPS, 1 type I PKS, 1 type 3 PKS, 3 terpene and 3 clusters of unknown type. SMURF returned 13 dimethylallyl tryptophan synthases (DMATS), 4 NRPS, 3 NRPS-Like, 3 PKS and 1 PKS-like clusters. A complete list can be found in Table 2. As reported by (Chen et al., 2014), *Exophiala dermatitidis* has fewer PKS and NRPS than *Aspergillus oryzae, Aspergillus fumigatus* or *Aspergillus nidulans* (Yu and Keller, 2005). It exhibits however 13 DMATS while the three *Aspergilli* cited above exhibit only 2,7 and 2 DMATS, respectively (Inglis et al., 2013). DMATS are catalyzing the first biosynthesis step of the ergot alkaloid (Tudzynski et al., 1999), a potent mycotoxin.

**Table 2:** **List of backbone genes in *Exophiala dermatitidis* involved in the production of secondary metabolites**.

**Gene**	**Type**	**Comment (fold, fdr)**
DMATS	HMPREF1120_00352	(4.11, 0.009)
	HMPREF1120_01260	(2.86, 0.026)
	HMPREF1120_01968	
	HMPREF1120_02200	
	HMPREF1120_06543	
	HMPREF1120_07933	
	HMPREF1120_08032	
	HMPREF1120_08132	
	HMPREF1120_09268	
	HMPREF1120_09269	
	HMPREF1120_08670	(3.55, 0.011)
	HMPREF1120_09038	(3.20, 0.008)
	HMPREF1120_09090	
NRPS	HMPREF1120_01440	
	HMPREF1120_02993	Acetylaranotin, toxin
	HMPREF1120_04809	
	HMPREF1120_06043	(7.511, 0.0045)
	HMPREF1120_07636	(2.46, 0.033)
NRPS-like	HMPREF1120_00598	(13.55, 0.005)
	HMPREF1120_03318	
	HMPREF1120_07093	
PKS	HMPREF1120_03173	
	HMPREF1120_06568	
	HMPREF1120_06570	t1PKS
	HMPREF1120_07394	t3PKS
PKS-Like	HMPREF1120_08091	
TERPENE	HMPREF1120_02863	
	HMPREF1120_03149	Terpene Cyclase (3.46, 0.012)
	HMPREF1120_09198	(3.11, 0.008)

Exophiala dermatitidis genome contains 13 DMATS, 8 NRPS/NRPS-like, 3 TERPENE and 8 PKS/PKS-like backbone genes. 4 DMATS, 2 terpene and 3 NRPS/NRPS-like backbone-enzymes were upregulated during skin invasion.

#### 3.7.6. Other pathways

Various cell wall genes are significantly (fdr < 0.05) regulated in the skin experiment. Chs1 (HMPREF1120_07981, 9.49), Phosphoacetylglucosamine mutase (HMPREF1120_02062, 4.38), Skt15 (activator of chitin synthase 3, HMPREF1120_06335, 10.32 ; HMPREF1120_05528, 2.33), Chia (Class III chitinase, HMPREF1120_03399, 14.03), Chitin deacetylase (HMPREF1120_08023, 5.11), Crh1 (transglycosidase, HMPREF1120_00627, 6.99), Mlg1 (mixed linked glucanases) (HMPREF1120_09051, 8.64), CelA (cellulose synthase, HMPREF1120_ 04699, 5.68) are upregulated. On the other hand N-acetyl-beta-glucosaminidase (HMPREF1120_06285, 0.23; HMPREF1120_06035, 0.27), HMPREF1120_01790 (UDP-N-acetylglucosamine 6-dehydrogenase, 0.26) and HMPREF1120_08078 (1,3-β-transglucosylases) are downregulated.

In *Exophiala dermatitidis* under skin condition, members of the HOG and Ca^2+^/Calcineurin pathways are strongly regulated, which might explain the significant regulation of cell wall and chitin-related genes, known also in *Candida albicans* (Lenardon et al., 2010).

Three light sensing genes were upregulated upon growth on skin, i.e., HMPREF1120_06318 (Wc-1, 3.72), HMPREF1120_00072 (VelB, 3.75) and HMPREF1120_07867 (Phy-1, 3.98). In contrast the carotenoid oxygenase (HMPREF1120_02864, 0.22) is downregulated.

## 4. Discussion

In this study an experimental setup for fungal growth on *ex-vivo* skin explants was established. To our knowledge this is the first time that *Exophiala dermatitidis* infection of *ex-vivo* skin models was successfully performed under experimental conditions in the lab. Based on this infection system, the transcriptome of *Exophiala dermatitidis* during its first contact with *ex-vivo* skin tissue was reproducibly sequenced. With the help of these transcriptome data, genes playing a role during the first contact of the fungus with the *ex-vivo* skin models were clearly identified. Further, state of the art bioinformatics pipelines for genomic annotation, functional annotation and differential expression analysis pipelines were designed for this study, allowing us to reproducibly investigate the sequencing data and genome data.

Based on this analysis, we could substantially increase the number of annotated protein-coding genes and found new homologs to ncRNAs, i.e., snR4, snR72, snR74. For the first time long non-coding transcripts were reported in the group of black yeasts. While it is not clear what the function of these transcripts could be, it is known that lncRNAs, as a cis-regulatory element, are able to control the transcription of the loci in their neighborhood (see Quinn and Chang, 2015 for a review). Chacko et al. (2015) showed that a long non-coding RNA, RZE1, regulates the yeast-to-hypha transition in *Cryptococcus neoformans*. Further in Kaposi sarcoma-associated herpes virus it was shown that the lncRNA β2.7 has a role in preventing stress response and apoptosis in the host cell (Amaral et al., 2013).

In line with the studies of Blasi et al. (2015), circular RNAs and fusion transcripts were detected, two kind of transcripts that were previously connected to infections in archaea and virus (Lau et al., 2014). We could also show that circRNAs and fusion-transcripts were also differentially regulated and seem therefore important for infection in *Exophiala dermatitidis*. The finding that various ncRNAs that are exported outside the cell in vesicles are also upregulated during skin infection, might indicate that they might be useful during the host infection, as was previously reported for *Botrytis cinerea* (Weiberg et al., 2013).

*Exophiala dermatitidis* grows successfully on and into the skin. This was supported both by the microscopic pictures (see Figure 1) and the functional enrichment analysis. The most significantly enriched gene functions are related to active replication and transcription such as MCM complex, which is the eukaryotic replicative helicase (Bochman and Schwacha, 2009), ribosome biogenesis, AAA+ ATPases (Ogura and Wilkinson, 2001), helicases and SF2-related N-terminal domain, which is involved in all aspects of RNA and DNA metabolism (Fairman-Williams et al., 2010). The genome *Exophiala dermatitidis* contains four adhesins, CalA, Ecm33, Car10, and Phr1 that were shown to successfully promote adhesin in either *Candida albicans* or *Aspergillus fumigatus* (de Groot et al., 2013). While none of them are significantly regulated, Phr1 is the 85th most highly expressed gene in the *Exophiala dermatitidis* transcriptome. In *Candida albicans* Phr1 is involved in adherence to laminin, murine lung cells (Upadhyay et al., 2009), abiotic surfaces and human epithelial cells (Calderon et al., 2010).

Seven out of eight homologs of *Candida albicans* Hsp70 (Ssa1), are either highly upregulated or highly expressed during fungal growth on skin. Hsp70 was shown to be involved in cell proliferation, cell death and morphological switch toward the yeast-form in *Paracoccidioides brasiliensis* (da Silva et al., 1999). Besides its role in morphology, Hsp70 was shown to be responsible for cell invasion in *Candida albicans* (Phan et al., 2007; Sun et al., 2010). While the yeast is prevalent in *Exophiala dermatitidis* during growth on the skin model (See Figure 1D), there are evidence from the upregulation of Hgc1 and Efg1, two genes that were previously connected to the hyphal growth (Dieterich et al., 2002; Mayer et al., 2013), that some cells in the experiment might be switching to the hyphal form. This discrepancy could be explained by the fact that *Exophiala dermatitidis* growing on and in the skin might adopt different morphologies. Due to the lack of biomass both populations were sequenced together.

The skin environment further triggers the upregulation of various secondary metabolites clusters. *Exophiala dermatitidis* exhibits a high number of DMATS compared to other filamentous fungi and 4 DMATS, 2 Terpene and 3 NRPS/NRPS-like clusters are upregulated while none of the backbone genes involved in the production of secondary-metabolites are downregulated. One of the upregulated NRPS cluster is a putative antibiotic precursor (HMPREF1120_06043) which might help the fungus to overcome the skin microbiota barrier. DMATS upregulation, which might be linked to an increased production of ergot alkaloid, might be used to attack skin cells. The cytotoxicity of ergot alkaloids was previously shown in human renal cell lines (Haarmann et al., 2005).

Beyond the known fungal virulence factors, *Exophiala dermatitidis* seems to employ mechanisms to infect and invade its host that were not previously reported. Apparently only pyomelanin, i.e., the melanin originating from the L-Tyrosine pathway, is upregulated during infection. In *Aspergillus fumigatus*, the production of pyomelanin was shown to be upregulated during infection but dispensable for virulence (Heinekamp et al., 2012). In the fungal pathogen *Sporothrix brasiliensis* pyomelanin was shown to protect this fungal pathogen against antifungal drugs (Almeida-Paes et al., 2016). Further melanin and genes involved in melanin production were shown to be crucial for iron uptake (Ding et al., 2014) in *Cryptococcus neoformans*. Similarly, in the bacterial pathogen *Legionella pneumophila* pyomelanin is involved in iron uptake (Zheng et al., 2013). We can hypothesize that the upregulation of pyomelanin in *Exophiala dermatitidis* during the skin model infection is probably connected to the upregulation of iron acquisition, similar to what is obeserved in *Cryptococcus neoformans*. The other melanin-production pathways were not regulated during the growth on the *ex-vivo* skin model. Inversely the L-Tyrosine pathway was not upregulated during salt, UV and ozon (24 ppm) stress (manuscript in preparation).

During the infection of the skin model, *Exophiala dermatitidis* experiences a complete reprogramming of its carbon metabolism. Snf1 (HMPREF1120_02538), which responds to declining level of glucose and is the central player in the glucose repression pathway in *Saccharomyces cerevisiae* (Usaite et al., 2009; Conrad et al., 2014), undergo a five-fold upregulation (fdr = 0.003) when *Exophiala dermatitidis* is growing on skin. Snf1 was shown to be crucial for the growth on any non-fermentable carbon source (Young et al., 2003). Eventhough glucose from the cell culture media is available during the artificial infection, it seems that *Exophiala dermatitidis* is at least in part switching to the gluconeogenesis pathway. It was previously shown that *Candida albicans* was also showing a switch to gluconeogenesis when internalized in macrophages (Lorenz et al., 2004). It can be seen through the upregulation of the Fox1,2 and Pot1 genes that the production of acetyl-CoA, which is needed for generation of malate from glyoxylate through the malate synthase (fc = 21, fdr = 0.003) in the glyoxylate cycle, is increased. Mdh1-3, which are needed in glyoxylate cycle for the production of oxaloacetate, are upregulated, and therefore lead to phosphoenolpyruvate, the substrate of the gluconeogenesis pathway. The upregulation of nearly all genes involved in the gluconeogenesis pathway indicates that phosphoenolpyruvate is actively converted into glucose (see Supplementary Table 1, Figure 5).

While the carbon acquisition metabolism was significantly regulated during the skin experiment, we could not clearly derive which genes were responsible for the nitrogen acquisition mechanism. Two protease families, i.e., classified as C26 and S16 in merops, were significantly enriched in the set of the upregulated genes. The S16 Lon protease family is a known bacterial and fungal virulence factor (Takaya et al., 2003; Breidenstein et al., 2012; Cui et al., 2015). It is unclear if keratin is actively degraded by *Exophiala dermatitidis*. Two members of the M03 and one member of the S8 merops class, were upregulated upon infection. Huang et al. (2015) showed that these types of proteases in combination with M28, which in our case was downregulated when *Exophiala dermatitidis* grew on skin, are able to degrade keratin. Ssu1, a sulfite efflux pump which plays a crucial role in the catabolism of keratin in dermatophytes (Grumbt et al., 2013), is downregulated in *Exophiala dermatitidis* during infection. HMPREF1120_05465, an aminoacid transporter protein is upregulated, while HMPREF1120_03271, an other aminoacid transportes is downregulated. HMPREF1120_06103, a peptide transporter, is strongly downregulated.

In order to sustain its accelerated growth on the skin the fungus is actively acquiring metals. Based on gene homology with genes reported in *Saccharomyces cerevisiae, Cryptococcus neoformans, Candida albicans* and *Aspergillus fumigatus* (Ding et al., 2014) we could determine that *Exophiala dermatitidis* is uptaking iron via the reductive iron uptake and the use of siderophores. In the reductive iron uptake pathway, Ftr1 and Fth1 are significantly upregulated indicating that this pathway might be used. *Exophiala dermatitidis* upregulates SidD, SidF, SidA and SidC, which are the enzymes responsible for siderophore synthesis (Chen et al., 2014). Further Sit1, a siderophore transporter,is upregulated (Heymann et al., 2002).

Other metal transporters upregulated are the magnesium transporters, namely HMPREF1120_04846, HMPREF1120_08107 and HMPREF1120_05571. This is in line with results of (Giles and Czuprynski, 2004), where it was shown that in *Blastomyces dermatitidis*, a dermatophyte, a lack of magnesium even for short period of time is detrimental to its proliferation. Transporters of zinc and related genes were found to be downregulated or not regulated in the skin environment which leads to the conclusion that zinc might not be crucial for the first week of infection.

Overall the improved annotation of coding and non-coding genes as well as the thorough quest for known virulence factors and mechanism from other fungal pathogens allows us to get a better insight into the virulence mechanism and adaptation of *Exophiala dermatitidis* during host invasion. Due to the restrained knowledge on this fungus, further studies are necessary in order to better apprehend the mechanisms in play during host infection by *Exophiala dermatitidis*. Based on this study, the first knock-out experiments using the Crispr/Cas9-System in *Exophiala dermatitidis*, are being conducted on various identified virulence factors in our lab.

## Author contributions

KS conceived the experiments and contributed the funding/consumables/tools. UM contributed the skin model. CP set up the skin experiments, BB developed the transcriptome sequencing. CP performed the experiments, EA did the TEM pictures. Development of bioinformatic tools for data analysis: HT. Data-analysis: HT, CP, and KS. Writing of paper: CP, HT, BB, UM, EA, KS.

## Funding

The work was supported by the VIBT-Extremophile Center. Equipment of the VIBT-Extremophile Center was used, financed by the BOKU-Equipment GesmbH.

### Conflict of interest statement

The authors declare that the research was conducted in the absence of any commercial or financial relationships that could be construed as a potential conflict of interest.

## Supplementary material

Supplementary Figure 1 **After 1 week, *Exophiala dermatitidis* grew through the wound and was visible on the undersurface of the skin model (Black spot in the middle)**.

Supplementary Figure 2 **Similarity assessment of the RNA sequencing runs with principal component analysis**. The clustering pattern fits well with the expectation from the experimental design.

Supplementary Table 1 **Summary of all results and raw data**.
