# Supplementary figures and images for: The Transcriptome of Exophiala dermatitidis during Ex-vivo Skin Model Infection
Source: Front Cell Infect Microbiol. 2016 Oct 24;6:136. doi: 10.3389/fcimb.2016.00136 (PMC5075926; doi:10.3389/fcimb.2016.00136)

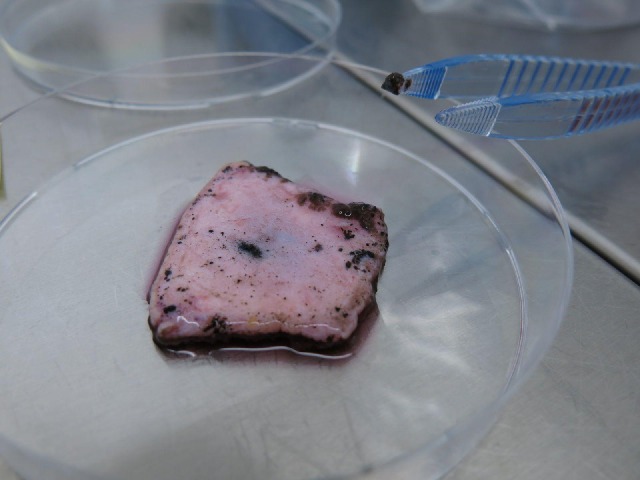

Supplement: Supplementary Figure 1 — After 1 week, Exophiala dermatitidis grew through the wound and was visible on the undersurface of the skin model (Black spot in the middle). [file Image1.JPEG]

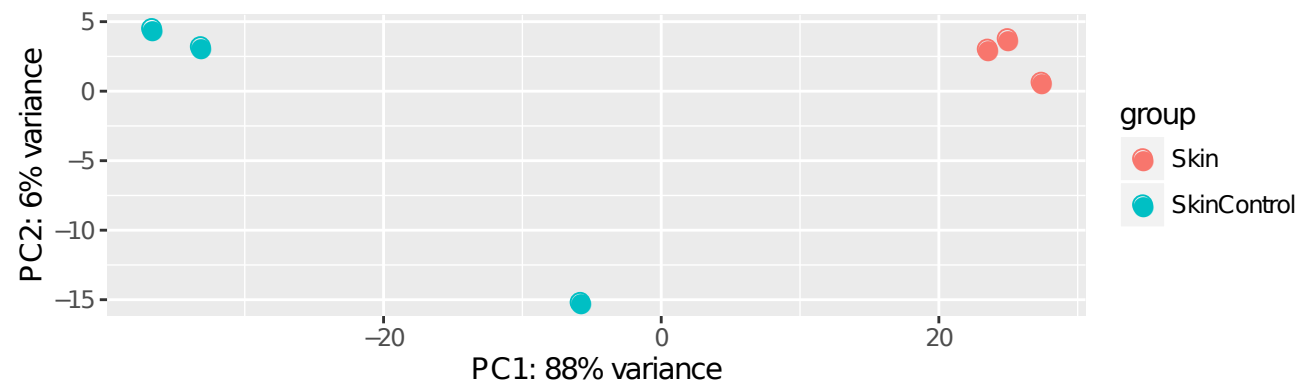

Supplement: Supplementary Figure 2 — Similarity assessment of the RNA sequencing runs with principal component analysis. The clustering pattern fits well with the expectation from the experimental design. [file Image2.PDF]
